# Community-Led Monitoring: When Community Data Drives Implementation Strategies

**DOI:** 10.1007/s11904-020-00521-2

**Published:** 2020-07-31

**Authors:** Solange Baptiste, Alain Manouan, Pedro Garcia, Helen Etya’ale, Tracy Swan, Wame Jallow

**Affiliations:** International Treatment Preparedness Coalition, Johannesburg, South Africa

**Keywords:** Community-led monitoring, Social accountability, HIV, Improving health service delivery, Community data

## Abstract

**Purpose of Review:**

Communities occupy a central position in effective health systems, notably through monitoring of health service quality and by giving recipients of care a voice. Our review identifies community-led monitoring mechanisms and best practices.

**Recent Findings:**

Implementation of community-led monitoring mechanisms improved service delivery at facility-level, health system-wide infrastructure and health outcomes among recipients of care. Successful models were community-led, collaborative, continuous and systematic, and incorporated advocacy and community education.

**Summary:**

Identifying and replicating successful community-led monitoring practices is a key pathway to equitable access to HIV and health services overall.

## Introduction

Although the value and impact of their work are often under-recognized, communities have an important role in and are essential to building and overseeing strong healthcare systems, as originally noted in the 1978 Alma Ata Declaration [1, 2]. Indeed, communities have already been active in ensuring accessibility, availability, and acceptability of health services [1, 3]. Since the beginning of the HIV epidemic, community action has been instrumental in securing political will and funding for HIV research, prevention, care, and treatment services [4, 5]. The contribution that communities make by monitoring healthcare and holding healthcare providers (HCP) and governments accountable for meeting their needs is particularly critical in resource-limited settings with weak healthcare and monitoring systems [6]. However, the contribution of community-led monitoring is not yet adequately recognised or maximised.

A common definition of “community” is groupings of people with shared interests, social interactions, behavioural norms, and/or geographical location [1, 7]. In this review, the term “community” refers specifically to intended end-users of health services, networks of people living with HIV (PLHIV), and civil society organisations working to promote rights-based access to care for key populations—those with the highest rates of and vulnerability to HIV, such as men who have sex with men (MSM,) sex workers, people who use drugs, and transgender persons. People who are members of these key populations are often excluded or discouraged from accessing services due to stigma, discrimination, and legal, economic, and other sociocultural barriers [5].

This review explores the evolving nature of community monitoring mechanisms in HIV and health overall, documents the contribution of community engagement to improving health service delivery and outcomes, and summarises implementation methods and best practices.

Community monitoring is a growing field; it demonstrates how community action and energy can be channelled to bring about change. To characterise this evolving field, a broad methodology was used: peer-reviewed articles, published from 2009 to date, were identified through a Pubmed and Google Scholar search using terms such as “community observatories”, “community-based monitoring”, “community-led monitoring”, “social accountability”, and “citizen monitoring”; articles on community monitoring in HIV and other health fields and their references were reviewed to identify additional articles, including grey literature.

## Summary of Findings

### Community Health Monitoring—a Literature Review

Public health observatories have emerged naturally, out of a legitimate need for systematic data collection and surveillance to monitor population health and health systems. Generally, without seeking to replace existing epidemiological and surveillance systems, observatories collate data from multiple sources to provide comprehensive overviews that support meaningful data interpetation and consequent national decision-making [8, 9]. While monitoring by public health observatories dating back to the 1970s has been documented [10], the notion that healthcare recipients can also generate data on the quality of health services is more recent. Often, accounts of community engagement have ranged around the lower end of the “ladder of participation” through token involvement or as passive actors [11] such as research participants. Whether the system in which they work is categorised as “social accountability”, “community-based monitoring”, “community-led monitoring”, and most recently “community observatories”, the recipients of healthcare services are more than passive recipients of care; instead, they are agents capable of holding health service providers accountable and advocating for optimal health services to meet their needs [12]. Communities experience health system failings such as unavailable or malfunctioning equipment, drug stockouts, stigma and discrimination, poor healthcare provider (HCP) attitudes, and inadequate infrastructure, but, in many settings, they lack the mechanisms or capacity to raise grievances due to cultural norms, power imbalances, and fear of reprisal [6, 13, 14]. Thus, community monitoring fills a gap where other systems and institutions are unable or unwilling to document shortfalls and rectify them.

### Types of Community Monitoring

The literature highlights the ways that different community monitoring mechanisms have increased coverage, equitable utilisation of health services, and responsiveness among site-level personnel and institutions. The key elements of each model are summarised in Table 1.

**Table 1:** A summary of community monitoring models found in the literature

Monitoring model	Monitoring mechanism (s)	Stakeholders involved	Sample outcomes and achievements
Health Facility Committees (HFCs)	• A joint committee of community and HCPs collects recipient of care grievances and works with HCPs to address them • Regular meetings between HFC members and healthcare providers/decision-makers track progress on the resolution of identified issues	• Healthcare providers • HFC members (community representatives and HCPs)	Implementation of HFCs in Kenya, Peru, and Zimbabwe led to [11]: • Increased use of health services and knowledge on health • Improved access for low-income people from reduced user fees • Greater uptake of antenatal care • Fewer cases of diarrhoea • More staffing and outreach services • Improved financial management
Citizen Report Cards (CRC)	• Metrics for a ‘report card’ are identified through phone interviews and surveys with recipients of care • A healthcare facility’s performance is compared to a national standard or a similar facility at externally facilitated meetings of recipients of care and HCPs	• Healthcare providers and decision-makers • Recipients of care • External facilitator (NGO, CSO)	Implementation of report cards in a Ugandan setting led to (15,17 •): • Higher rates of child immunisations • Decrease in mortality rates among children under age 5 • Reduced health care provider absenteeism • Reduced waiting • Higher outpatient use • Greater cleanliness
Community Score Cards (CSC)	• Communities and HCP develop indicators separately, then agree on a plan for corrective action • Progress on the indicators is jointly monitored by healthcare providers and communities in biannual meetings • A variation of this methodology is the use of health advocates, who devise action plans to address recipient of care grievances and work with healthcare providers or MOH officials to address them, and track outcomes and resolutions.	• Healthcare providers • End-users or CHWs	Application of a CSC at a Malawi site led to [19]: • Improvement in provider-community relationships • Increased responsiveness to recipient of care needs by healthcare providers • Increased male involvement in maternal/ newborn health and family planning • Greater access to reproductive health information • Higher rates of youth engagement in services Action by health advocates led to: • Less tardiness among HCPs • 50% increase in daily prenatal exams • Speedier ART initiation for persons co-infected with HIV/TB • Expansion of mobile clinics and their services (immunisation, family planning, chronic disease management) • Infrastructure upgrades (improved toilet facilities, a separate unit for TB patients)
Community Treatment/Health Observatories	• Systematic, regular collection of quantitative (monthly) and qualitative (quarterly) data by community and recipients of care networks using indicators identified through a pilot or baseline assessment • Data are analysed and discussed in multi-stakeholder meetings, where advocacy plans are developed, implemented and tracked • In another formulation, recipients of services or HCWs observe gaps in quality and access at facilities and report back to a community health observatory.	• Recipients of care, community health workers, community and civil society organisations • Health care providers • Health ministry officials and policy-makers • Academic institutions	Implemented in 11 countries across West Africa, the CTO model led to (22, 23 •): • Reduced incidence of drug and lab reagent stockouts • Increased uptake of differentiated ART service delivery models • Improved HIV treatment monitoring • Revision of site-level data tracking mechanism and greater use of RVLT results in treatment monitoring • Increased rates of HIV testing among key populations and young people CHOs implemented in West Africa led to: •Shorter waiting times at facilities, reduced stockouts and replacement of malfunctioning equipment

*Health facility committees* (HFC) are composed of HCPs and community representatives and act as a liaison between them. HFCs collect healthcare recipient grievances, review them in regular meetings, act on them, and provide feedback to communities. Taken together, installation of HFCs at health facilities in Kenya, Peru, and Zimbabwe increased the utilisation of health services by 20%, including antenatal care, and health-related knowledge among recipients of care; it improved access for low-income recipients of care by reducing user fees and lowered rates of diarrhoea. In addition, HFCs enabled increases in staffing and outreach services and they identified and addressed financial mismanagement [11].

*Citizen report cards* track the quality of health services based on metrics that communities identify and prioritise. These metrics are collected through phone or in-person surveys. Progress on metrics is measured against either a national standard or performance by other health facilities and monitored during facilitated meetings between HCPs and communities [6, 15]. The implementation of report cards in a Ugandan setting led to higher child immunisation rates, decreases in mortality rates among children under age 5, reduced health care provider absenteeism, shortened waiting lines, and increased outpatient use and cleanliness of the facilities [15, 17•]. Furthermore, where communities were educated on their rights and on local diseases, health outcomes improved and were still evident after a 3-year period—reduced drug stockouts, increased pre- and post-natal care, increased knowledge on tuberculosis (TB), reduced HIV stigma, and increased utilisation of services [16].

*Community Score Cards* (CSC) track the performance of health systems using indicators that are developed by and jointly agreed upon by community members and HCP. They are translated into an action plan during an interface meeting. Implementation and progress on the action plan are jointly assessed by HCPs and communities in biannual meetings [18]. In Malawi, the application of a CSC led to significant improvements along select metrics: relationship between providers and recipients of care (37%), increased male involvement in maternal newborn health and family planning (33%), as well as access to reproductive health information (22%), and increased youth engagement in reproductive health services (23%) [19].

Feinglass et al. [14] describe the operations of “defensores de saude” or *health advocates* in Mozambique, who support communities in addressing health system failures as they arise. Health advocates educate communities on health policy standards and their rights in accessible language and collect data on their complaints, including demographics of beneficiaries of care, actions taken, and resulting outcomes. They identify problems, devise solutions, and establish a timeline for corrective action with HCPs, working either amicably at the site level, or by escalating up the health ministry decision-making ladder, if needed. Working with health advocates led to improvements in tardiness among healthcare providers, a 50% increase in daily prenatal exams, speedier ART initiation for persons co-infected with HIV and tuberculosis (TB), and expansion of mobile clinics and the types of services offered at peripheral sites, such as immunzations, family planning, and chronic disease management. Changes to the infrastructure, such as better toilet facilities and a separate unit for TB patients, were also implemented.

*Community treatment observatories* (CTO) involve the systematic and regular collection of quantitative data across the HIV prevention, care, and treatment cascade. Observatories work via formal, written agreements between a health facility and community and other civil society networks. Community representatives are trained in health science and Monitoring and evaluation (M&E) methods and gather data across pre-established indicators, on a monthly basis—directly from health facility records and with support from health facility workers. This data is supplemented by qualitative data, collected on a quarterly basis; recipients of care respond to and open-ended questions linked to specific indicators. Data from the onset of community observatories are the baseline against which new data are compared with, and trends identified. This database can identify gaps in access to and quality of care and provide key evidence to support targeted advocacy [20].

The cornerstones of the community observatory model are data collection and analysis and regular review meetings with communities, health service providers, academicians, and national stakeholders where advocacy plans and concrete actions are devised [20]. This model has been implemented in 11 countries across West Africa.^1^ Within 18 months of the community treatment observatory (CTO) implementation, there was a 8.4% decrease in ART stockouts and a 10.7% decrease in lab reagent stockouts for viral load testing [21•]. Over the same period, CTO implementation resulted in 23,618 more people initiated on ART, 16,844 more viral load tests performed, a 29% increase in viral suppression rates and an increased average quality of care rating (from 3.8 to 4.2 out of 5) across all monitored health sites [21•]. In a year of implementation of the CTO in Sierra Leone, HIV testing increased among men who have sex with men (85%), female sex workers (100%), people who inject drugs (96%), pregnant women (90%), and young people (90%) while ART uptake increased by 93% among people living with HIV [22]. Evidence of low uptake of HIV services among key populations obtained from Sierra Leone’s national CTO informed the development of a national Differentiated Service Delivery (DSD) policy thus reducing barriers and increasing the convenience and efficiency of HIV clinics for recipients of care. Elsewhere, CTO implementation led to better treatment monitoring for people on ART in Mali and revised site-level data tracking mechanisms in Gambia [23•]. CTO data strengthened the intensity of community advocacy in Malawi by highlighting irregular routine viral load testing (RVLT) access at monitored sites and contributed to the alignment of national RVLT guidelines with the WHO recommendations [24] (from 24-month intervals to 12-month intervals [25].

Combining elements of the CSC and CTO approaches, health monitors (or “sentinelles”) in francophone Africa who were recipients of care or community health workers, collaborated within a community health observatory (CHO) structure to report on the quality of services at health facilities through SMS, smartphone applications or in-person meetings with community observatory facilitators. The activities of a CHO in Burkina Faso to shorter waiting times, less frequent drug stockouts, and a replacement of old, malfunctioning equipment at facilities in Burkina Faso, Guinea, and Niger. Another CHO in Burkina Faso highlighted that pregnant women were being wrongfully asked to pay user fees and the situation rectified [26].

## Lessons Learnt from the Literature

### Leveraging Opportunities from Community Monitoring

Communities can play a catalytic role in improving health outcomes, service delivery, and overall quality of life and understanding of health within communities. Since communities are at the heart of the approach and stand to benefit from improved access to and quality of health services, they have ownership and are empowered to take an active role in their health—and healthcare. The involvement of recipients of care—and other affected communities—in monitoring their health systems is essential. They have insights and knowledge that are unavailable to external actors and can provide feedback on the acceptability of solutions—or actual solutions—to resolve issues that they have identified [27]. This involvement goes far beyond a watchdog role; it creates and shows the power of a collaborative approach, as communities fill an important gap [20, 26] with an impact extending beyond the health facility. Thanks to community-derived evidence, for instance, ART adherence clubs in South Africa that were considered ineffective were found not to be operating; evidence without which their contribution would have been dismissed [27]. Elsewhere, CTO data collectors in Ghana sensitized religious leaders, women’s groups and chiefs on HIV and stigma against key population groups [21•].

The foundation of these models is their transparency and accountability. Community monitoring is inclusive of all sections of the population, including the most marginalised, who are usually excluded—women, youth, people living in poverty, and key populations [5, 11]. The literature shows the value and impact of community accountability and monitoring processes for improving health outcomes in HIV and in other health fields. In addition, community monitoring could address additional issues of social consequence e.g. climate change [28] and corruption [29]. When technology literacy and its infrastructure are adequately addressed, information and communication technologies (ICT) can be leveraged to reinforce and strengthen community-led monitoring processes (faster data collection, aggregation, analysis, data visualisation, and geo-mapping) [30].

### Operational Challenges of Community Monitoring

Community-led monitoring requires financial resources and technical expertise, as essential for enabling and capacitating communities with knowledge of the key issues relevant to, and processes for community monitoring [31]. Political will and buy-in of HCP and site-level authorities are also necessary for adequate corrective measures when grievances require policy development, enforcement, or a more systemic solution [6, 7].

## Community Monitoring: Lessons Learnt from Implementation

The literature review supports observations from ITPC’s implementation of community treatment observatories in West and Southern Africa, where these key components were essential to achieving positive outcomes:

- *Community-led*: As per UNAIDS, “community-led responses are actions and strategies that seek to improve the health and human rights of their constituencies, that are specifically informed and implemented by and for communities themselves and the organizations, groups, and networks that represent them.” [32•]. Rather than top-down approaches where communities are simply consulted, communities themselves drive the agenda [11, 20]. When communities operate without external influence (e.g. political), they have ownership and decision-making authority, and they are more effective [12]. Community ownership ensures representative and inclusive mechanisms. The CTOs in West Africa were hosted by networks of people living with HIV, who were attuned to the needs of recipients of care, and felt ownership in generating evidence to address existing barriers to life-saving HIV prevention, testing, treatment, and care services [33]. The fact that people who are members of key populations (KP) in Gambia were data collectors reduced fear and encouraged other members of KPs to access services [21•].
- *Continuous and systemic*: Beyond addressing discrete incidents, CTOs identify trends and systemic deficiencies and advocate for change. In documenting stockouts at monitored health facilities, the CTO in Senegal highlighted deeper inefficiencies, motivating the country’s central pharmacy to review its dispatching system to accelerate supplies to health facilities [33]. Similarly, the health ministry in Malawi extended opening hours at all public health facilities in response to findings from the CTO which identified standard opening hours, at monitored sites, as a barrier to HIV and RVLT services for truck drivers and sex workers [25].
- *Collaborative*: Successful outcomes from community monitoring mechanisms incorporated a platform for exchange and collaborative problem-solving between communities, HCPs, and other stakeholders; the CTO community consultative groups engaged health ministry representatives and academics [20]. This platform strengthened relationships between parties for mutual support and joint ownership of solutions. In Mali, for instance, the national network of PLHIV collaborated with pharmacists at target facilities to revise the data systems for patient follow-up. Additionally, the academic collaboration with CTOs across West Africa gave the data credibility and acceptability to the results at national, regional, and international levels [33].
- *Community education*: A strong capacity-building component ensures data collectors and CTO host organisations are strengthened through the tailored trainings and capacity assessments that are part of the implementation process [33]. Data collectors, who were trained in HIV science and monitoring and evaluation methods, generated and analysed high-quality evidence that gained the attention of and were acted on by health facilities and national governments [21•]. As a result of CTO implementation, a community network in Zambia was integrated into the national DSD technical working group and invited to present at the national HIV/AIDS conference [25]. Recognition of the importance of CTO data and monitoring mechanisms also led Malawi and Zimbabwe to integrate the model into their 2020–2025 National Strategic Plans for HIV and AIDS, and into Malawi’s PEPFAR’s Country Operational Plan 2020 [25]. Empowered communities empower their peers: in Guinea Conakry, data collectors educated key populations and young people on their rights and the services they, as recipients of care, were entitled to—at a health facility [21•]
- *Advocacy*: Creating demand for services and community mobilisation was critical to achievement of positive outcomes from community monitoring. Identifying the failings of health systems is insufficient unless appropriate action is taken to remedy them [32•]. Since the onset of the HIV epidemic, the contribution of community activism to increased treatment access and coverage has been undeniable [7] and needs to be maintained. Evidence-based advocacy by community-based organisations and networks of people living with HIV sustained improvements in access to services across the HIV cascade in West Africa [21•–23•]. In Cote d’Ivoire, user fees were eliminated after evidence-based advocacy by the CTO identified them as a barrier to health service access [33].

Taken together, insights from the literature as well as from practical CTO implementation reveal what community-led monitoring is—and what it is not. Community monitoring highlights issues and brings them to those who can act upon them; it is a systematic and continuous process that seeks to bring about sustainable change. Other community-led accountability and monitoring mechanisms such as alert systems [33], suggestion boxes, patient rights charters, cross-sectional surveys, and community hearings with HCP can be effective in improving service delivery [3], but they are not necessarily monitoring interventions. Additionally, recipients of care are the drivers of community-led monitoring and, as such, community monitoring does not include the monitoring of recipients of care *by* HCPs, nor does it include programmatic M&E systems that include specific community-centred indicators.

## Conclusion

While the notion of health monitoring is not new, community monitoring—where recipients of care themselves monitor the quality of health services—is an evolving field. Communities can fill in gaps, verify national- and/or site-level accounts of health service quality, and ensure that services meet the needs of recipients of care. Communities are entitled to know their rights and to have an active role in ensuring that they are being upheld [14].

Although this review shows that community monitoring mechanisms exist in different forms, including health facility committees, community scorecards, citizen report cards, health advocates, and community treatment/health observatories, further research could establish clear standards and precise terms that differentiate monitoring from other effective and necessary community-led interventions. The literature review demonstrates how community monitoring methods have improved health outcomes (better maternal and child health increased immunization rates), health infrastructure (building renovations, equipment), and health care provider behaviour (more responsiveness, friendlier attitudes, reduced tardiness and absenteeism). However, external factors such as adequate financial and technical support, political will, and consideration of socio-political realities are vital to sustain such improvements. These observations are aligned with evidence from the implementation of ITPC’s community treatment observatories in Western and Southern Africa. Successful outcomes resulted from the key components of community treatment observatories—that they are community-led, continuous and systematic, collaborative, and incorporate elements of community education and evidence-based advocacy. The adoption of CTO methodology in national policy and decision-making platforms is confirmation of their contribution to improving health outcomes for recipients of care, facility-level delivery, and national health policy and systems. Additional research could identify an optimally effective monitoring frequency and the cost-effectiveness of community monitoring.

## Data Availability

N/A

## Footnotes

1 Benin, Cote d’Ivoire, Gambia, Ghana, Guinea, Guinea-Bissau, Liberia, Mali, Senegal, Sierra Leone, Togo
